# Psychosocial Work Environment Among Musicians and in the General Workforce in Norway

**DOI:** 10.3389/fpsyg.2020.01315

**Published:** 2020-06-26

**Authors:** Anna Détári, Hauke Egermann, Ottar Bjerkeset, Jonas Vaag

**Affiliations:** ^1^York Music Psychology Group, Department of Music, University of York, York, United Kingdom; ^2^Faculty of Nursing and Health Sciences, Nord University, Bodø, Norway

**Keywords:** musicians, psychosocial work environment, musicians’ health, epidemiological study, genre, role differences

## Abstract

Musicians suffer from physical and mental health symptoms more frequently than the general population. Although their specific demands and challenges have been researched increasingly in the past, explanations still remain somewhat unclear. We use a large epidemiological data set to compare psychosocial work environment among 1,607 members of the Norwegian Musician’s Union with a national sample of 8,517 employees from the general Norwegian workforce. Musicians reported more control over their work; however, they felt less supported and acknowledged, had more work-family conflicts and less motivation, and perceived their work as more demanding compared to the general workforce. In the musician sample, results indicated that classical and contemporary musicians are experiencing a less favorable psychosocial environment in terms of control, demands, and acknowledgment, orchestral players felt less control and soloist less support. Future studies should explore possible interventions to improve musicians’ psychosocial work environment.

## Introduction

Psychosocial work environment has been a widely researched topic in the recent decades (Regulies, 2019). Reaching back to the 1980s (Alfredsson et al., 1982), the term has been described as the interaction between the individuals’ personal experiences and the characteristics of the workplace. Recently, the first handbook of psychosocial epidemiology was published (Kiwimaki et al., 2017) systematically addressing psychosocial factors as contributors to illnesses and health, discussing interventions and policies, and setting a direction for future research. The book is an extensive summary of the growing body of research that shows that the impact working conditions can have on health is considerable. Psychosocial factors have been linked to different health problems, such as ischemic heart disease (Eller et al., 2008) and coronary heart disease (Peter and Siegrist, 2000), and to cardiovascular mortality in general (Johnson et al., 1996). Moreover, there is evidence that it contributes to musculoskeletal pain (Torp et al., 2001) especially back pain (Hoogendoorn et al., 2000), and there are also links to obesity (Jääskeläinen et al., 2015), the prevalence of smoking (Brisson et al., 2000), and mental health problems (Stansfeld and Candy, 2006; Harvey et al., 2017), such as burnout (Lindblom et al., 2006; Aronsson et al., 2017), and depression (Bonde, 2008; Theorell et al., 2015).

Interestingly, musicians seem to be especially vulnerable to some of these health problems. Research has shown that they struggle with high prevalence rates of musculoskeletal injuries and pain (Zaza, 1998; Kok et al., 2016). Depending on the examined population, and the definition of “musculoskeletal injury” and “musculoskeletal pain,” different percentages are reported, but as an example, 77–87% of orchestral musicians (Leaver et al., 2011; Ackermann et al., 2012; Berque et al., 2016), 67% of college-level music students (Stanek et al., 2017), and 23–93% of pianists suffer from performance-related musculoskeletal problems (Bragge et al., 2006).

Mental health problems also occur more frequently in musicians compared to the general population. One study found that 23% of orchestral players suffered from symptoms of depression, and 33% of social phobia (Kenny et al., 2014), and symptoms of both depression and anxiety was two times higher than the general workforce (GW) in another sample (Vaag et al., 2016). The higher level of psychological distress was also linked to the higher prevalence of sleep disorders in the population (Saksvik-Lehouillier et al., 2017; Aalberg et al., 2019), and in addition to that, musicians also tend to suffer from work-associated hearing loss, which is related to increased levels of sound exposure (Backus and Williamon, 2009; Schmidt et al., 2014, 2019).

For some of these factors, especially the musculoskeletal problems, the physical demands of instrumental playing are held responsible. Asymmetrical body postures, non-ergonomic instruments, and repetitive movements are only a few physical aspects of instrumental practice that can directly be linked to injuries (Kaufman-Cohen and Ratzon, 2011; Bird, 2013). The high levels of psychological distress are usually explained by public exposure and performance anxiety. However, there is not enough evidence that these factors are exclusively responsible for the wide variety of problems that musicians are experiencing in their lives and careers. There are also other factors, originating from the unique working environment that are associated with the profession, such as working long hours, late nights, extreme concentration levels, traveling, insufficient equipment, or performance spaces, which also negatively impact this population (Jacukowicz, 2016).

In spite of the established links between physical and mental health issues and the psychosocial work environment in the GW, very few studies have investigated the psychosocial work environment of musicians before. Akel and Duger (2007) studied 90 music students playing three different instruments in higher education and found that certain psychosocial factors, such as “job demand,” “physical extortion,” and “physical load” were associated with stress and musculoskeletal injuries. However, these findings were not compared to the general population. A similar study focused entirely on how the psychosocial work environment influences the symptoms of stress in Danish orchestras, and found lower job satisfaction, higher emotional demands, lower social support, lower influence, and a lower sense of community compared to the GW (Holst et al., 2011). Self-reported hearing loss, mental health issues, and stress factors in orchestral musicians were linked to a poorer psychosocial work environment (Hasson et al., 2009); moreover, a review also showed its connections to musculoskeletal injuries (Jacukowicz, 2016).

The literature is scarce, and as we can see from the examples, it is restricted not only to the classical genre but also one type of role musicians can fulfill: orchestral playing. This biased sample is common not only in these psychosocial studies but is typical across the literature investigating pain, injuries, mental health issues, and other problems affecting musicians. The bulk of the literature draws conclusions from samples consisting exclusively of classical orchestral musicians, very often not specifying that their findings are informative only in this one population (Kok et al., 2016). Playing different genres of music and fulfilling different roles within an orchestra, band, or group might have a significant effect on the musicians’ experience, their work environment, and health. Different genres are often performed in different environments, require different education and workload, and have a different schedule and support system. Moreover, being a soloist or front figure fundamentally differs from being part of a large band or orchestra in terms of public exposure, demands, and psychosocial stressors.

The psychosocial work environment is most frequently measured with one of three models. The demands, control, support model (JDC-S; Karasek and Theorell, 1990) examines the intensity and quantity of the work, the exercised authority in one’s work, and the level of support the worker receives from the workplace and colleagues. This model has been developed further by Bakker and Demerouti (2007) by adding other characteristics of the work environment, such as the relationship between resources and demands. The third model is the effort-reward model (ERI), which was proposed by Siegrist (1996), who argued that the experience of imbalance between the effort of the worker and the received reward can lead to serious stress because it violates the reciprocity in an important area of life. When developing tools to measure the psychosocial work environment, these models were taken into consideration, which resulted in valid and reliable instruments, such as the General Nordic Questionnaire for Psychological and Social Factors at Work (QPS-Nordic scale; Wannström et al., 2009), and the Job Content Questionnaire (JCQ; Karasek et al., 1998).

In conclusion, very little is known about the psychosocial work environment of musicians. The existing studies have small sample sizes; they are examining very specific subpopulations and most often are not comparing their finding to the general population (Kok et al., 2016). In order to fill this gap in the literature, a large-scale epidemiological study was conducted based on a diverse sample of Norwegian musicians.

Our research questions have been the following:

1. Does musicians’ psychosocial work environment differ from the general workforce?
2. Are there significant psychosocial differences between musicians performing in different genres?
3. Are there significant differences in the psychosocial work environment of musicians performing in different roles?

Musicians suffer from a wide variety of physical and mental problems. In the recent decades, there has been a growing interest in exploring the possible contributing factors. Examining the influence of the psychosocial work environment of musicians compared to the general population might help us to understand the most important differences between musicians and non-musicians. In addition, by measuring differences between players of different genres, and musicians working in different settings, we can pinpoint the most problematic settings, understand their characteristics, and can help to form theories on how these psychosocial factors might affect the individuals. This would enable us to design interventions and new policies in the future to make meaningful changes in the work environment of this vulnerable population.

## Materials and Methods

### Participants and Setting

#### Musician Sample

In 2013, 4,168 members of the Norwegian Musician’s Union were invited to take part in a survey, of which 2,121 responded (51%) [1,016 female (47.9%), 1,105 male; mean age, 44.5 years, SD = 10.7]. All musicians who worked professionally in the 12 months prior to the questionnaire were selected to participate, 1,607 musicians in total. Informed consent of participation to the project was given online before answering the anonymous questionnaire. Ethical approval for this research project was given by the Norwegian Committees for Medical and Health Research Ethics.

#### Control Sample (“General Workforce”)

The control sample is based on the Norwegian Survey of Level of Living. In the survey, a total randomized sample of 20,460 subjects (17–66 years) were invited to participate. Data collection were undertaken between June 2009 and January 2010, using telephone interviews. A total of 12,255 participated in the survey (61%). Of these, 8,517 (70% of the eligible) participants were currently employed and were listed with an International Standard Classification of Occupations (ISCO) code and constituted the workforce sample in this study; 4,182 were women (49%) and 4,336 were men. The mean age was 42.2 years (SD = 12.7).

### Materials

#### Psychosocial Work Environment

In order to compare our sample of musicians to the GW, psychosocial work environment was measured based on the questions used in the Norwegian Surveys of Level of Living, which again was based on selected items from the JCQ and the QPS-Nordic questionnaire (Wannström et al., 2009). The variables that were covered by the Level of Living survey were the following: *job control*, *job demands*, *social support*, *effort–reward* (salary and acknowledgment), *work–family conflict*, and job motivation, and thus also included in the survey of musicians.

*Job control* was measured, using a 5-point Likert scale, using four items (α = 0.76) measuring skill discretion (e.g., “To what degree can you decide your own working pace?”), and decision latitude (e.g., “To what degree can you influence decisions which are important to your work?”).

*Job demands* were measured by asking respondents to respond on a Likert scale from 1 (very rarely or never) to 5 (very often or always) to three items (α = 0.75). One example of the items was “How often is it necessary to work at a fast pace?”

*Social support* was measured on a Likert scale with one item phrased as following, “If you need it, how often do you get support and help in your work from your work colleagues?” Due to a large degree of freelancers in our sample, we decided to not include an additional item measuring support from the leader (e.g., conductor).

*Effort–reward* was measured with two items assessing the discrepancy between perceived effort and reward in terms of salary and acknowledgment (e.g., “The size of my salary is appropriate compared to my efforts and achievements at work”).

*Work–family conflict* and *job motivation* were measured on a 5-point Likert scale using the following items: “How often do the demands at work disturb your family-life?” and “How often do you feel motivated and engaged in your work?”

#### Demographics

In addition to sex and age, we collected information on role as a musician (solo/front figure, member of ensemble/band, or orchestra), as well as genre types (according to Spellemannsprisen, the Norwegian music awards).

#### Statistics

Statistical analysis of descriptives and different forms of analyses of variance [ANCOVA and multivariate analysis of covariance (MANCOVA)] were conducted in order to look at differences between musicians and other workers on psychosocial work environment variables. Analyses were conducted using IBM SPSS 25.0.

## Results

### Musicians and the General Workforce

The results of a between-subjects MANCOVA show a significant difference between the psychosocial work environment of musicians and the GW in all seven outcome variables (see Table 1). In this analysis, we also controlled for potentially covarying demographic differences by introducing the variables age and gender as additional factors and covariates into the model. Table 2 presents means of psychosocial work environment variables separated by participant groups. Musicians report to have more control over their amount of work and experience fewer disturbances. However, they rated the demands they have to fulfill higher than the GW, received less support, and felt less rewarded both in terms of salary and acknowledgment. Moreover, they experienced more conflict between their work and family life and were less motivated in their work.

**TABLE 1:** *F* test results for testing differences between musicians and the general workforce on psychosocial work environment variables.

Dependent variable	*F*	*df*1, *df*2	*p*	*η^2^*
Control	1,641.130	1, 9,994	<0.001	0.141
Demands	1,835.488	1, 9,994	<0.001	0.155
Support	832.420	1, 9,994	<0.001	0.077
Reward–salary	149.053	1, 9,994	<0.001	0.015
Reward–acknowledgment	800.097	1, 9,994	<0.001	0.074
Work–family conflict	935.928	1, 9,994	<0.001	0.086
Job motivation	74.209	1, 9,994	<0.001	0.007

*Sex and age were used as control variables (not shown).*

**TABLE 2:** Mean table showing differences between musicians and the general workforce on psychosocial work environment variables.

Dependent variables	General workforce *M* (SD)	Musicians *M* (SD)
Control	2.57 (0.81)	3.44 (0.86)
Demands	2.47 (0.57)	3.18 (0.79)
Support	4.29 (0.95)	3.53 (1.03)
Effort–reward–salary	3.37 (1.40)	2.94 (1.26)
Effort–reward–acknowledgment	4.32 (0.98)	3.55 (1.15)
Work–family conflict	2.15 (1.18)	3.12 (1.02)
Job motivation	4.34 (0.86)	4.15 (0.75)

### Genres and Roles

Participants were asked to indicate the genre they perform most frequently out of 15 different choices, which were the following: pop, jazz, contemporary music, rock, blues, country, metal, electronic music, hip-hop, folk music (or traditional music), dance orchestra, classical music, show music, music for children, or other. Due to the limited number of respondents in some of the categories, only pop, rock, jazz, contemporary, folk music, show music, classical music, and music for children were separately analyzed, and the remaining categories (blues, country, metal, electronic, hip-hop, and dance orchestra) were placed in the “other” category. In addition, participants were asked to specify the roles they were most frequently performing or associated with (soloist, playing as the member of a small ensemble, being an orchestral player, or other). We found a significant association between the two variables genre and role [Table 3, χ^2^(24) = 390.8, *p* < 0.001, Cramers’s *V* = 0.285]. It is very likely that these associations stem from the specific characteristics of each genre, especially the size of the group they are generally performed in. As an example, unsurprisingly large orchestras are very common in the classical genre but nearly non-existent in rock music.

**TABLE 3:** Crosstabulation of variables genres and roles.

	Pop	Jazz	Contemporary	Rock	Folk music	Classical	Norwegian folk	Children’s music	Other	Total
Soloist/front figure (*n* = 397)	25%	21%	27%	22%	49%	22%	40%	21%	30%	25%
Ensemble/band (*n* = 734)	64%	71%	55%	71%	44%	32%	49%	42%	52%	46%
Orchestra (*n* = 298)	2%	3%	4%	0%	1%	36%	0%	2%	4%	19%
Other (*n* = 178)	9%	6%	14%	7%	6%	10%	12%	35%	15%	11%
Total%	100%	100%	100%	100%	100%	100%	100%	100%	100%	100%
Total *n*	106	180	49	83	72	764	33	52	268	1,607

Since both participant variables (genre and roles) were associated with each other, we entered these variables simultaneous as factors in MANCOVA models that subsequently tested if both factors are significantly associated with each of the seven work environment variables (Table 4).

**TABLE 4:** Influence of musician’s most performed genre and role on psychosocial work environment variables.

Factor	*F*	*df*1, *df*2	*p*	*η_p_*^2^
**Dependent variable: control**				
Genre	7.502	8, 1,596	<0.001	0.036
Role	78.688	3, 1,596	<0.001	0.129
**Dependent variable: demands**				
Genre	3.498	8, 1,596	0.001	0.017
Role	7.356	3, 1,596	<0.001	0.014
**Dependent variable: support**				
Genre	0.527	8, 1,596	0.837	0.003
Role	5.531	3, 1,596	0.001	0.010
**Dependent variable: reward–salary**				
Genre	3.362	8, 1,596	0.001	0.017
Role	1.245	3, 1,596	0.292	0.002
**Dependent variable: reward–acknowledgment**				
Genre	1.267	8, 1,596	0.257	0.006
Role	1.686	3, 1,596	0.168	0.001
**Dependent variable: work–family conflicts**				
Genre	1.896	8, 1,596	0.057	0.009
Role	1.276	3, 1,596	0.281	0.001
**Dependent variable: job motivation**				
Genre	1.893	8, 1,596	0.057	0.009
Role	1.451	3, 1,596	0.226	0.003

*Sex and age were used as control variables (not shown).*

**FIGURE 1:**
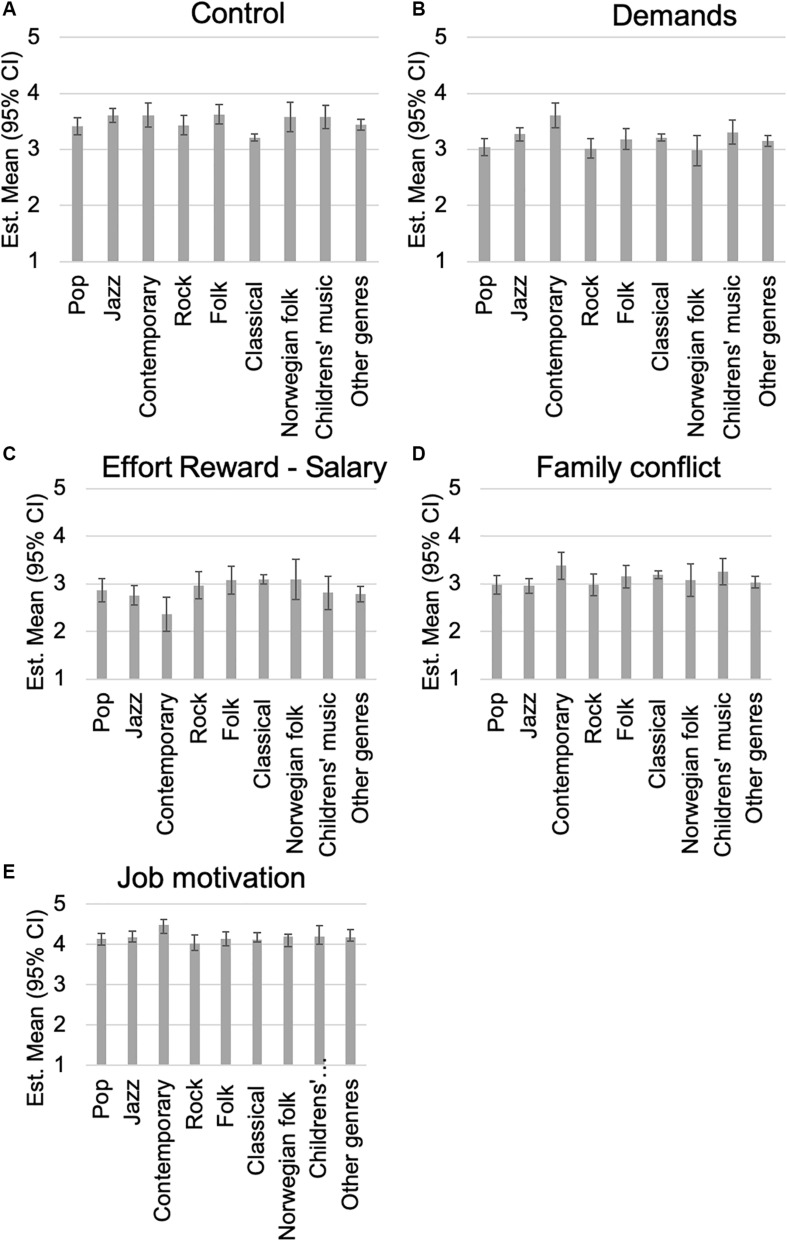
Estimated marginal means with 95% of **(A–E)** selected work environment variables separated by most frequently performed music genre.

In terms of control over the working conditions, classical musicians scored significantly lower than all the other genres (see Figure 1A, *p* < 0.05). From the remaining six variables, four were associated with music genres, and all of these took place between performers of contemporary music and all the other measured groups. Contemporary musicians not only reported more demands (Figure 1B, *p* < 0.05), felt less rewarded in terms of salary (Figure 1C, *p* < 0.05), more work–family conflict (Figure 1D, non-sig. trend *p* < 0.10) but also had higher levels of job motivation (Figure 1E, non-sig. trend *p* < 0.10).

**FIGURE 2:**
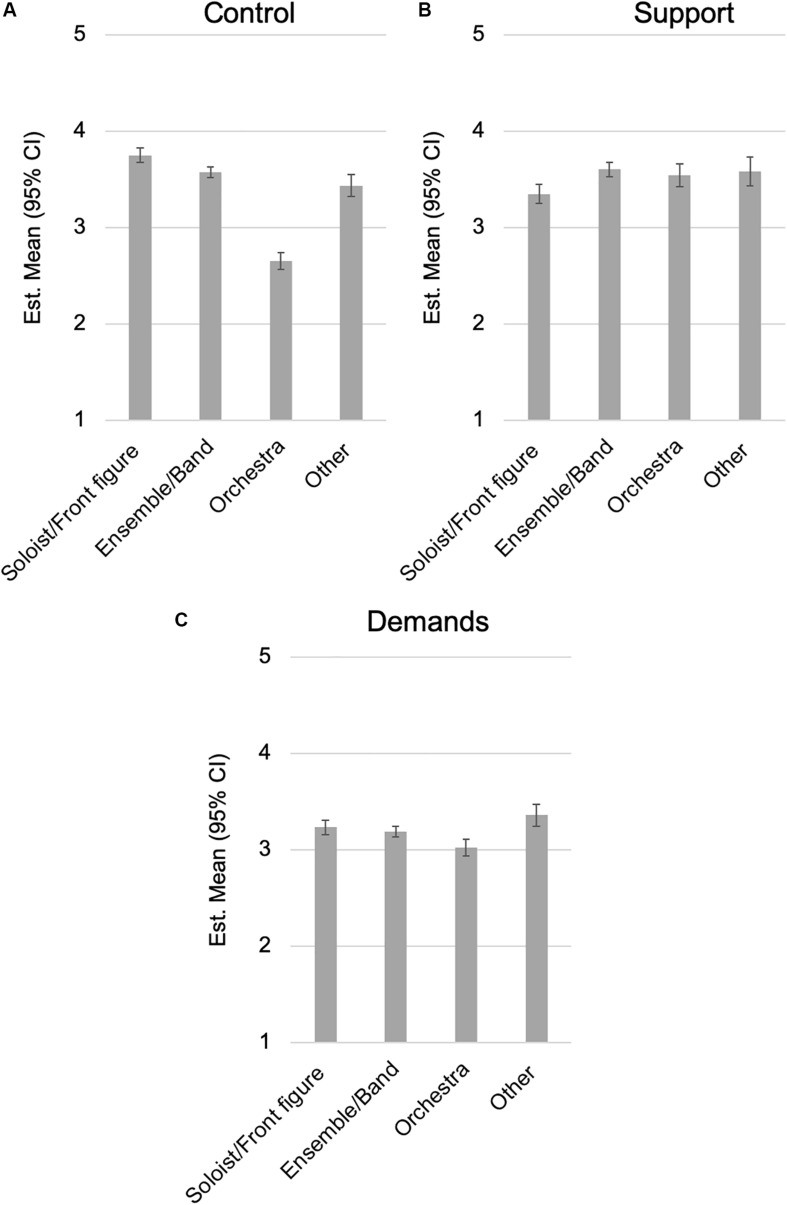
Estimated marginal means with 95% of **(A–C)** selected work environment variables separated by role.

Performing in different roles (soloist, playing as the member of a small ensemble, or being an orchestral player) affected the psychosocial work environment. The MANCOVA showed that playing as a member of a larger, orchestral constellation significantly lowered the control the participants had over their work (Figure 2A). Moreover, soloists and front figures felt least supported among these groups (Figure 2B), and the group of musicians who indicated “other” experienced more demands in their work (Figure 2C).

## Discussion

The results show that musicians’ psychosocial work environment significantly differs from the GW in all psychosocial aspects, and there are significant differences between groups playing different genres and fulfilling different roles within the music community. The variables are going to be discussed one by one.

### Control

The findings showed that musicians reported having more control over their work compared to the GW, which suggests a better psychosocial environment. The questions associated with this variable were measuring the extent to which the person is autonomous in the decisions concerning their work, more specifically, the autonomy of choosing the task, the scheduling of the work, and the methods used. These characteristics line up with the Job Demand–Control–Support model (JDCS) by Johnson et al. (1989), which is often used to measure psychological demand and occupational health (Canjuga et al., 2010).

However, when examining differences between genres and roles, we found that classical musicians and large orchestral players have less control than the rest of the sample. This interesting overlap between two cross-sections of the data can be explained by the traditions of performing classical music where large symphonic orchestras play a major role. The lack of job control might be attributed to playing under the close supervision and command of the conductor whose aim is to create a balanced, uniform sound (Allmendinger et al., 1996). Therefore, the musicians have to adjust their playing accordingly to fit in, losing some of their personal expression and unique approach to the material; in other words, a clash in their collective and individual musical aim might appear (Gaunt and Dobson, 2014). In addition to that, playing classical music creates more constraints for the player: there are usually no improvised structures and is less freedom in interpretation (Altenmüller and Jabusch, 2010); moreover, the notated sounds have to be performed in the right pitch, with the right articulation and dynamics, and exactly at the right moment.

These characteristics are linked to the core nature of orchestral playing and classical music performing, so seemingly there is no way to drastically change it in order to improve the psychosocial work environment. However, there might be small ways to give some freedom to the players; for example, allow them to influence the choice of repertoire, or give them more musical freedom in the interpretation of the material.

### Demands

The special demands of the musical profession have been the topic of research due to the frequent prevalence of occupational diseases. However, most of the research is on physical demands such as the repetitive movements, asymmetric seating, or psychological ones, such as performance anxiety (Jacukowicz, 2016). Psychosocial demands might be another source of the problem, and as the findings show, the musicians reported much higher demands than the GW.

The questions associated with this variable covered workload, and the need to work from home—which, in case of a musician, would mean practicing their instrument and learning new material. Musicians need preparation time at home—the quantity largely depending on the material they are playing—in order to keep their ability fresh and master the music they are supposed to play. Moreover, musicians who compose, score, or orchestrate their own music need additional time for this creative work. These are hidden, additional demands a musician meets during their work.

The most vulnerable group in terms of demands seem to be the musicians whose repertoire mostly contains contemporary music. This might be attributed to the special instrumental techniques and challenging notations associated with contemporary music, which are most often not part of the basic curriculum (Johnson, 2005), are more demanding to read and execute, and need more practice (Castellengo et al., 1993; Alberman, 2005). In addition, the contemporary music scene—by its nature—is constantly producing new pieces, which means that these performers have to play new repertoire more often.

### Support

Musicians reported lower levels of support than the GW, with soloist and front figures scoring lowest within the musician population. This finding is especially unfavorable to the music population since social support was shown to reduce occupational stress and anxiety by addressing stress-producing environmental circumstances (SPECs; Beehr and McGrath, 1992). In other words, it can buffer the effect of the job strain and demands (Bakker and Demerouti, 2007). The questions specifically addressed the support from colleagues, aiming to examine musicians as a social group.

This particular approach might be the reason for the front figures and soloist to report lower levels of support. In classical performance, soloists often work with many different ensembles, which prevents them to form stable working relationships with the musicians they are performing with. The same can be stated about some jazz soloists and pop and rock musicians who not always perform with the same band.

In addition, since being a solo performer is also coming with more public exposure, and as a result, more pressure, this could mean that there is a higher need for support in this population.

### Reward (Salary and Acknowledgment)

The findings showed that musicians found that their work is less rewarded, both in terms of salary and acknowledgment. These two variables are closely associated; however, they might express very different problems. The financial reward for engaging in music often comes from the performances themselves and, in the case of orchestras and bands, the rehearsals. However, as mentioned under “demands,” the musicians already need to master the material they play prior to rehearsals, and this personal work is very rarely acknowledged financially. In all the other professions musicians have been compared to, it is typical to have fixed work hours, and the individual is not expected to prepare for hours for the next working day. In addition, sacrificing weekends and evenings for rehearsals and concerts might not be reflected in the amount the musicians receive.

The financial reward for one’s work comes from either the organization (e.g., orchestra, band), managers, or self-created freelancing opportunities. As the data show, large orchestral players are more likely to be content with their salary than soloist or ensemble players—so it might be possible that the stability of the income, as well as the amount, is a factor influencing this particular construct. However, musicians seem to value certain aspects of the entrepreneurship, and as one exploratory study shows, many lead successful freelancing careers (Coulson, 2012).

In terms of being acknowledged, the questions were aimed at acknowledgment coming from peers and colleges. This particular type of acknowledgment differs from the one coming from an audience, which is an entirely different construct with both positive and negative factors (Brand et al., 2012). Peers share similar instrumental abilities and went through similar training; therefore, they are less likely to endorse their colleagues as the audience. Unfortunately, the literature on peer support between musicians is scarce, and most examined the social constructs in music conservatoires (Papageorgi et al., 2010), where peer support was found an important tool to meet the psychological demands of the performance, which could improve the psychosocial work environment. On the other hand, the high levels of perfectionism—which has been repeatedly found in the general music population—is linked to competitiveness (Flett et al., 1994), which might result in less peer acknowledgment.

### Work–Family Conflicts

The next outcome variable describing the psychosocial work environment was work–family conflicts. This subdimension describes how much the personal and professional lives interfered with each other in the case of musicians and the GW. Our findings showed that musicians have significantly more conflicts, and within the musicians’ group, contemporary players tended to struggle with more problems than musicians who played other genres. There were no significant differences between the musicians fulfilling different roles.

One of the possible reasons why musicians have a more conflicting work–family life is the time schedule of their work. Most concerts take place in the evenings, requiring performing musicians to stay up and work late (Jacukowicz, 2016) and adjust to irregular working patterns. Additionally, many of them also work in teaching positions, which is often an integral part of a musician’s life (Mills, 2004), and adds to the workload.

This sporadic schedule of musicians can be compared to shift workers. Shift work is defined as work undertaken in “non-standard” hours, late evening or night hours, work on weekends, and irregular working hours in general (Costa, 2003). Shift work has serious health consequences (Costa, 1997), including heart diseases and digestive disorders, but family-life conflicts seem to be one of the most obvious risks. Being out of synchronization with the rest of society, meaning that their work, leisure, and sleep times are skewed compared to the general population, can make it challenging to organize a day (Costa, 1997), can corrupt social well-being (Costa, 2003), and negatively impact interpersonal relationships.

### Job Motivation

One of the most surprising findings was that musicians’ job motivation was slightly lower than the GW. Musicians are generally viewed as people who have chosen their passion as their profession, creating a rather romantic notion of the job. Indeed, the motivation to engage with music on a professional level is more associated with intrinsic motivation, a satisfaction gained from the music itself (Hallam, 2011), and is centered around the development of a musical identity, a “musical self.” Improvement on the instrument and development of social connections, success, and enjoyment were reported as the positive key elements of building this self-concept and the motivation to decide to have a music career (Schnare et al., 2011).

Given the extreme amount of dedicated work musicians have to complete in order to become a professional (Ericsson, 2008), intrinsic motivation, which stems from personal interest and enjoyment (Ryan and Deci, 2000), and leads to high-quality learning, seems crucial. Musicians draw personal satisfaction and fulfilment from playing music (Hallam, 2011), and there are also social rewards that are offered to a music performer, such as the interaction with the audience; yet, the results here indicate that this intense motivation might get somewhat lost once performance is used to make a living.

Dobrow (2013) studied the dynamics of “calling,” which can be understood as the motivation to do meaningful work, which was previously viewed as a stable, unchangeable contrast (Bunderson and Thompson, 2009). However, she proposes a more dynamic model based on the longitudinal data from musicians, presenting evidence that it can fluctuate—most often decline—in correlation with ability, behavioral involvement, and social comfort relating to the activity. These characteristics seem to be linked to psychosocial factors; therefore, the “dynamic calling” model offers a possible explanation to the low job motivation presented in the sample.

In terms of genres and roles, one significant difference was shown in the sample: contemporary musicians reported more motivation than the rest of the musician sample.

## Limitations and Conclusion

### Limitations

Self-report bias is one of the most commonly discussed limitations of questionnaires (Razavi, 2001). Extreme or central tendency in responding, negative affectivity bias, socially desirable responses, and acquiescence are a few examples of response behaviors that might affect the data. In our particular sample, the bias of the conscious or unconscious desire to exaggerate one’s perceived problems could be present due to the frequently experienced and reported mental and physical health issues. While we acknowledge the existence of any possible response bias in this data collection, we assume that this might have been equally present for all groups studied, meaning that musicians versus GW or musicians of different genres and roles were all biased to the same degree. Since this bias would act as a constant factor influencing all groups in an equal way, it would be still be possible to test for group differences in an unbiased way.

While using objective measures, like experimentally manipulating the work environment, is proposed to solve the problem (Ganster and Schaubroeck, 1991), these might not capture the individual variations and subjective experiences. Since the target of the inquiry, the perceived psychosocial work environment, is subjective by nature, these objective experimental tools might not be appropriate to measure the construct. Some researchers promote methods that are using the report of external observers, such as peers, subordinates, or supervisors (Conway and Huffcutt, 1997). In the case of musicians’ psychosocial work environment, these methods might result in more reliable data; therefore, it is suggested for further research.

Our study is based on comparison between musicians and the GW, and we have based our design of questionnaire on a population study. This also involves the limitation of including single-item measures of some of the psychosocial variables. Another limitation is the age of our dataset of musicians (2013) and GW (2010). Nevertheless, due to a lack of previous studies, our results provide results that are important to further investigate in large samples.

## Conclusion

Decades of literature examined the high prevalence of musculoskeletal injuries in musicians, and more recently, the frequent mental health problems have been also placed in the center of attention (Zaza, 1998; Kok et al., 2016; Vaag et al., 2016). Until now, the origins of these problems have been sought in the physical demands of the work—including the posture and repetitive movements—and public exposure.

However, research in other areas shows that the psychosocial characteristics of a work environment can also significantly contribute to various health risks. In spite of this rich literature, the psychosocial work environment is largely understudied in the case of musicians. The findings of this paper show the differences between the general population and musicians and between musicians playing different genres and performing in different roles.

Given the links between the psychosocial work environment and health, and the frequent occupational diseases musicians are suffering from, understanding the work experience of this diverse population is crucial. It has been shown in other fields that interventions are effective tools to improve the psychosocial work environment and have long-term benefits for individuals (Bourbonnais et al., 2011). Future studies should develop similar interventions for musicians working in different settings, which can have further implications for their overall health.

## Data Availability Statement

The datasets generated for this study will not be made publicly available since at the start of the project publishing data was not included in the ethics form, the authors are unable to share raw data without approval. We can provide the following: making the items used in the questionnaires available for interested readers; making the development of indexes and procedures of statistical analyses available for the readers; providing the readers with links to the databases which we have drawn our control population from; giving a more thorough description of the population of musicians. Requests to access the datasets should be addressed to the corresponding authors.

## Ethics Statement

The studies involving human participants were reviewed and approved by Norwegian Committees for Medical and Health Research Ethics. The participants gave informed consent by continuing to participate in the study after reading the information provided.

## Author Contributions

OB and JV collected the data. HE and AD suggested the types of analysis to be carried out. The SPSS outputs were created by JV and analyzed by HE and AD. The Introduction, Discussion, and Conclusion sections were written by AD, the Methods and Materials and the Result sections were a collaborative work of all researchers. All authors contributed to the final manuscript.

## Conflict of Interest

The authors declare that the research was conducted in the absence of any commercial or financial relationships that could be construed as a potential conflict of interest.
